# Effect of HNO_3_ concentration on a novel silica-based adsorbent for separating Pd(II) from simulated high level liquid waste

**DOI:** 10.1038/s41598-017-11879-6

**Published:** 2017-09-12

**Authors:** Guo Ge, Xu Yuanlai, Yang Xinxin, Wang Fen, Zhou Fang, Yu Junxia, Chi Ruan

**Affiliations:** Key Laboratory for Green Chemical Process of Ministry of Education, Hubei Key Laboratory of Novel Reactor and Green Chemical Technology, Wuhan Institute of Technology, Wuhan, 430073 China

## Abstract

A new kind of silica-based (Crea + TODGA)/SiO_2_-P adsorbent with high selectivity adsorption for palladium (Pd) was synthesized to examined the applicability for partitioning process of high level liquid waste (HLLW). Adsorption behavior of Pd(II) towards (Crea + TODGA)/SiO_2_-P adsorbent and stability of adsorbent against HNO_3_ solution were investigated by batch method. The degradation parts of (Crea + TODGA)/SiO_2_-P dissolved in liquid phase were estimated by total organic carbon (TOC) analyzer. (Crea + TODGA)/SiO_2_-P adsorbent showed good selectivity adsorption for Pd(II) and reached equilibrium within 24 hr. The adsorption ability of (Crea + TODGA)/SiO_2_-P for Pd(II) and the content of TOC leaked decreased with the increasing of HNO_3_ concentration. In 3 M HNO_3_, the average of *K*_d_ values were 85.03 cm^3^/g and 26.10 cm^3^/g after contact time one to 28 days at 298 K and 323 K, respectively. While the content of TOC leaked from the adsorbent after 28 days were 1095 ppm (298 K) and 2989 ppm (323 K), respectively. Therefore, the adsorbent showed good stability at 298 K after contact with nitric acid for a long time. All results indicated (Crea + TODGA)/SiO_2_-P can be proposed as an applicable and efficient absorbent for separation of Pd(II) in 3 M HNO_3_ at 298 K.

## Introduction

For a sustainable development of nuclear energy, partitioning methods for the high level liquid waste (HLLW) generated from spent fuel reprocessing process have been studied worldwide. To strengthen nuclear waste management and minimize radioactive accumulation, separation of some fission product element (FPs) from HLLW is much more desirable^1, 2^. The platinum group metals (PGMs) are significant fission product elements in the HLLW including palladium, ruthenium and rhodium. Their supply and demand are becoming more and more imbalance because of the limited natural reserves. For the utilization of large amounts of PGMs in HLLW (~5.6 kg/1 t HU,UO_2_ fuel, 45 GWd/t)^3, 4^, palladium whose nuclides are stable or weakly radioactive can be widely used in electric, chemical, petroleum and pharmaceuticals industry^5, 6^. Therefore, it is necessary to separate palladium (Pd) from HLLW.

For this purpose, several extractants such as α-benzoin oxime (ABO)^7^, tertiary and quaternary^8^, dioctylsulphide^9^, bis-(2-ethylhexyl) sulphoxide (BESO)^10^, trialkylphoshinesulphide (TIPS)^11^, benzoyl-methylenetriphenylphosphorane (BMTTP)^12^, N,N,N′,N′-tetra-(2-ethylhexyl) thiodiglycolamide T(2EH)TDGA^13^, N,N′-dimethyl-N,N′-di-n-octyl-thiodiglycolamide (MOTDGA) and Tri-n-octulamine (TOA)^14^ have been developed for separation of palladium(II) from HLLW. But these ligands have various limitations, such as slow kinetics^9, 11, 12^, poor pH sensitivity^8^, solubility^12^, instability in acidic medium^9, 11^ and weak under irradiation^13^. To overcome these problems and simplify operation in real partitioning plant, there is a need for development of newer ligands with improved extraction efficiency.

Crea (N′-N′-di-n-hexyl-thiodiglycolamide) is a chelating extraction agent which is rarely reported in the literature before 2011^15^. Extractant Crea is supposed to have a strong affinity to palladium which is developed by put hexyl into N′-N′-dimethyl-N′-N′- di-n-octyl-thiodiglycolamide molecule. N,N,N′,N′-tetraoctyl-3-oxapentane-1,5-diamide (TODGA) has extensive application in the spent fuel reprocessing process because of its low water-solubility, good radiation stability, low cost and simple synthetic methods^16^. It showed much superior extraction ability for trivalent actinides and lanthanides^17^. In our previous work, (Crea + TOA)/SiO_2_-P adsorbent has excellent adsorption to Pd(II) and Pd(II) can be eluted out with Tu-0.1 M HNO_3_ (Tu: NH_2_CSNH_2_) solution^18^. Meanwhile, TODGA/SiO_2_-P adsorbent has adsorption affinity to REs (rare earths) but no affinity to Ru(III)/Rh(III), in which Ru(III)/Rh(III) group can be flowed out with feed solution and separated from REs group (REs group have similar property to minor actinide)^2^. Therefore, if Crea and TODGA extractants using together as synergistic extractants, Pd(II), Ru(III), Rh(III) can be eluted out by only one chromatography column and separated from other FPs group through different washing and eluted solutions.

Therefore, a new kind of silica-based adsorbent, (Crea + TODGA)/SiO_2_-P, was prepared in this study by impregnating Crea and TODGA two extractants into a macroreticular styrene-divinylbenzene copolymer which were immobilized in porous silica particles with a diameter of 50 μm. And adsorption properties of (Crea + TODGA)/SiO_2_-P adsorbent have been researched in 3 M (mol/dm^3^) HNO_3_ solution containing 15 mM Pd(II) at 298 K. To examine stability of (Crea + TODGA)/SiO_2_-P extraction resin against nitric acid solution at 298 K and 323 K, acid resistant behavior of (Crea + TODGA)/SiO_2_-P adsorbent has been studied by batch experiments and calculated by the concentration of carbon leaked into liquid phase from (Crea + TODGA)/SiO_2_-P adsorbent.

## Experimental

### Chemicals

**Figure 1:**
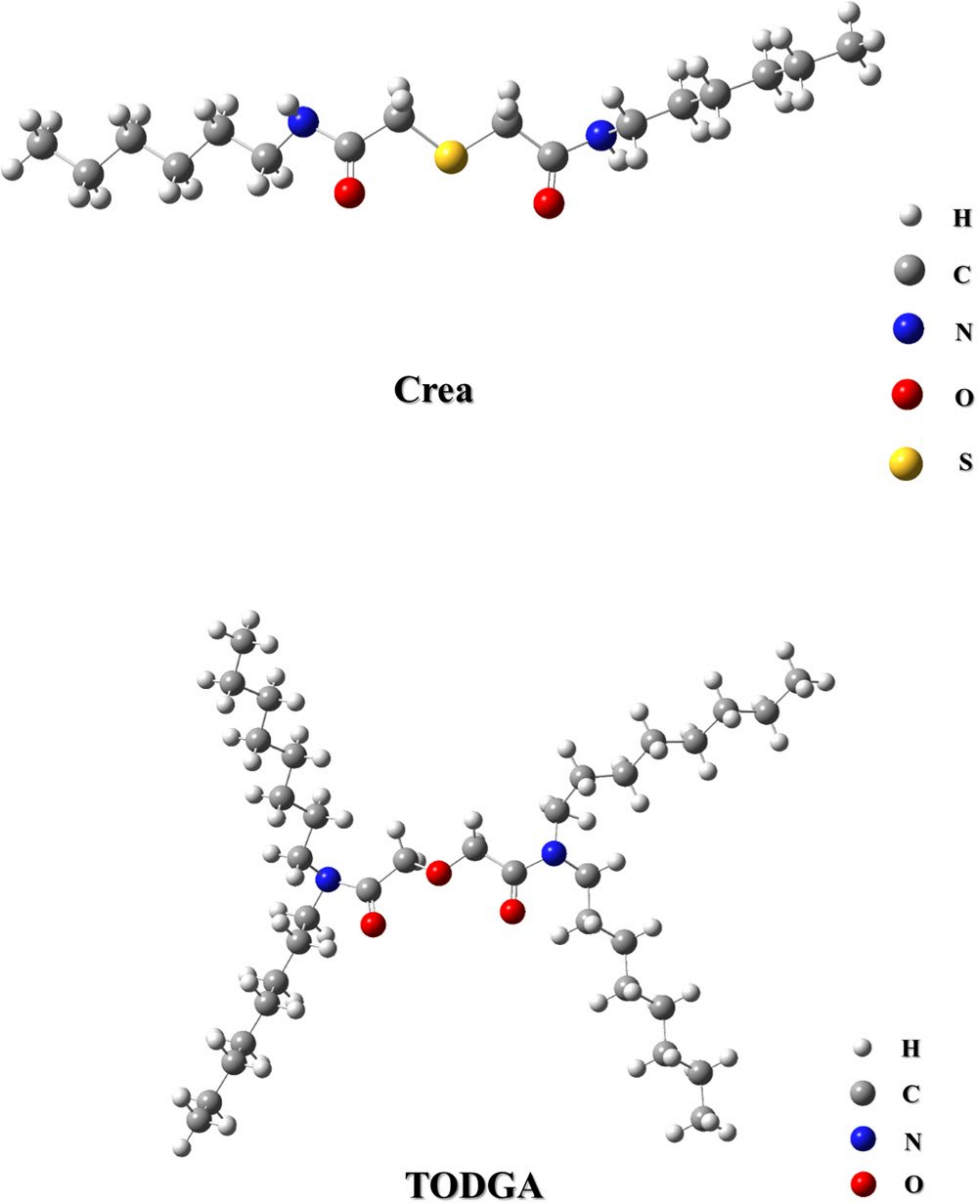
Chemical structural formula of Crea and TODGA two extractants.

All chemicals used were of analytical grade. The concentration of Pd(II) and HNO_3_ solution used were 15 mM and 0.01–5 M in batch experiment. Extractants Crea and TODGA, without any further purification, were purchased from Wako Pure Chemical Industries, Inc. and Kanto Chemical Co., respectively. The chemical structural formula of Crea and TODGA two extractants are shown in Figure 1 ^16, 18^. The SiO_2_-P particles fabricated were used as the support material according to a previous report^19^. The used (Crea + TODGA)/SiO_2_-P extraction resin was synthesized by impregnating and immobilizing Crea and TODGA extractants into the SiO_2_-P support particles with diameter size ranging of 40–60 μm.

### Preparation and characterization of (Crea + TODGA)/SiO_2_-P adsorbent

The SiO_2_-P particles were synthesized according to a previous study. It is a spherical silica particle with a diameter of 40 to 60 µm, a mean pore size of 600 nm, and a pore fraction of 0.69^19, 20^. “P” in the SiO_2_-P means styrene-divinylbenzene (SDB) copolymer immobilized inside the porous SiO_2_ by polymerization. The extractants Crea and TODGA were impregnated and immobilized into the pores the SiO_2_-P particles by using capillary effect and molecular inter-atomic forces.

The specific preparation method of (Crea + TODGA)/SiO_2_-P adsorbent is as follows: SiO_2_-P particles were washed by methanol three times and then dried in vacuum oven at around 313 K over 10 hr which can increase the affinity of SiO_2_-P particles with extractant molecules. Then a certain quantity of two chelating extractants, Crea and TODGA whose molar ratio was 2:1, dissolved by 280 cm^3^ of dichloromethane as a diluent in conical flask. Subsequently, 20.0 g of the activated SiO_2_-P particles were added into conical flask and the mixture was rotated and stirred evenly for 120 min at room temperature. Then methanol was removed at 323 K under reduced pressure by a rotary evaporator. The physical permeation and chemical polymerization took place inside the pores of the SiO_2_-P particles. After drying the residue in vacuum oven overnight at 313 K, the (Crea + TODGA)/SiO_2_-P adsorbent was obtained. The interaction between extractants (Crea + TODGA) and styrene-divinylbenzene (SDB) copolymer which was in the SiO_2_-P particles was considered as the intermolecular forces, i.e., van der Waals forces, which can hold non- or weak-polar molecules together.

The microstructure of (Crea + TODGA)/SiO_2_-P adsorbent was observed by scanning electron microscopy (SEM, Hitachi S-3100H) and transmission electron microscope (TEM, Hitachi HT7700). Thermal stability of (Crea + TODGA)/SiO_2_-P adsorbent was evaluated by thermal gravimetry and differential thermal analysis (TG-DTA, Shimadzu DTG-60) at the operating temperature range from 298–873 K in O_2_ atmosphere with a heating rate of 1 °C/min

### Adsorption equilibrium experiment

In the batch adsorption experiments, a weighed quantity of the silica-based adsorbent was mixed with 3.0 M HNO_3_ solution containing 15 mM Pd(II). The ratio of solid phase to liquid phase was 0.1 g to 5 cm^3^. Then the mixture was shaken at 160 rpm for different contact time. After the phase separation by vacuum filtration, concentrations of Pd(II) in the separated solution were analyzed by AAS (Thermo Scientific SOLAAR.M6). The distribution coefficient (*K*_d_, cm^3^/g) in the adsorption process was calculated as follows:

1 $${{K}}_{d}=\frac{{{C}}_{0}-{{C}}_{t}}{{{C}}_{{t}}}\times \frac{{V}}{{m}}$$

where *C*_0_ and *C*_t_ are initial and residual metal ion concentrations in liquid phase in mg/dm^3^ (ppm). *V* is the volume of solution in cm^3^ and *m* is the weight of adsorbent in g.

### Batch adsorption behavior in nitric acid solution

Effects of HNO_3_ on the adsorption behavior of palladium (II) were studied by batch adsorption method in different concentrations of HNO_3_ solution. The applied concentrations of HNO_3_ were 0.01–5 M and the contact time was up to 28 days. 0.1 g of dry extraction resin and 5 cm^3^ of HNO_3_ solution containing 15 mM Pd(II) were mixed in a glass vial and shaken at 298 K and 323 K, respectively. After the phase separation through vacuum filtration, the concentrations of Pd(II) in the liquid phase was determined by AAS (Thermo Scientific SOLAAR.M6).

### Degradation of (Crea + TODGA)/SiO_2_-P adsorbent

The degradation of (Crea + TODGA)/SiO_2_-P adsorbent was estimated by focusing on the elution of extractants. After batch adsorption experiments, the liquid phase was separated from the solid phase and dissolved organic carbon in liquid phase was measured by total organic carbon TOC analyzer (Shimadzu TOC-V_CPN_). All dissolved carbon was regards as the carbon of adsorbent.

The measurements were conducted at three different locations on experimental conditions, the average values and standard deviations were reported.

## Results and Discussion

### Characterization of adsorbent

**Figure 2:**
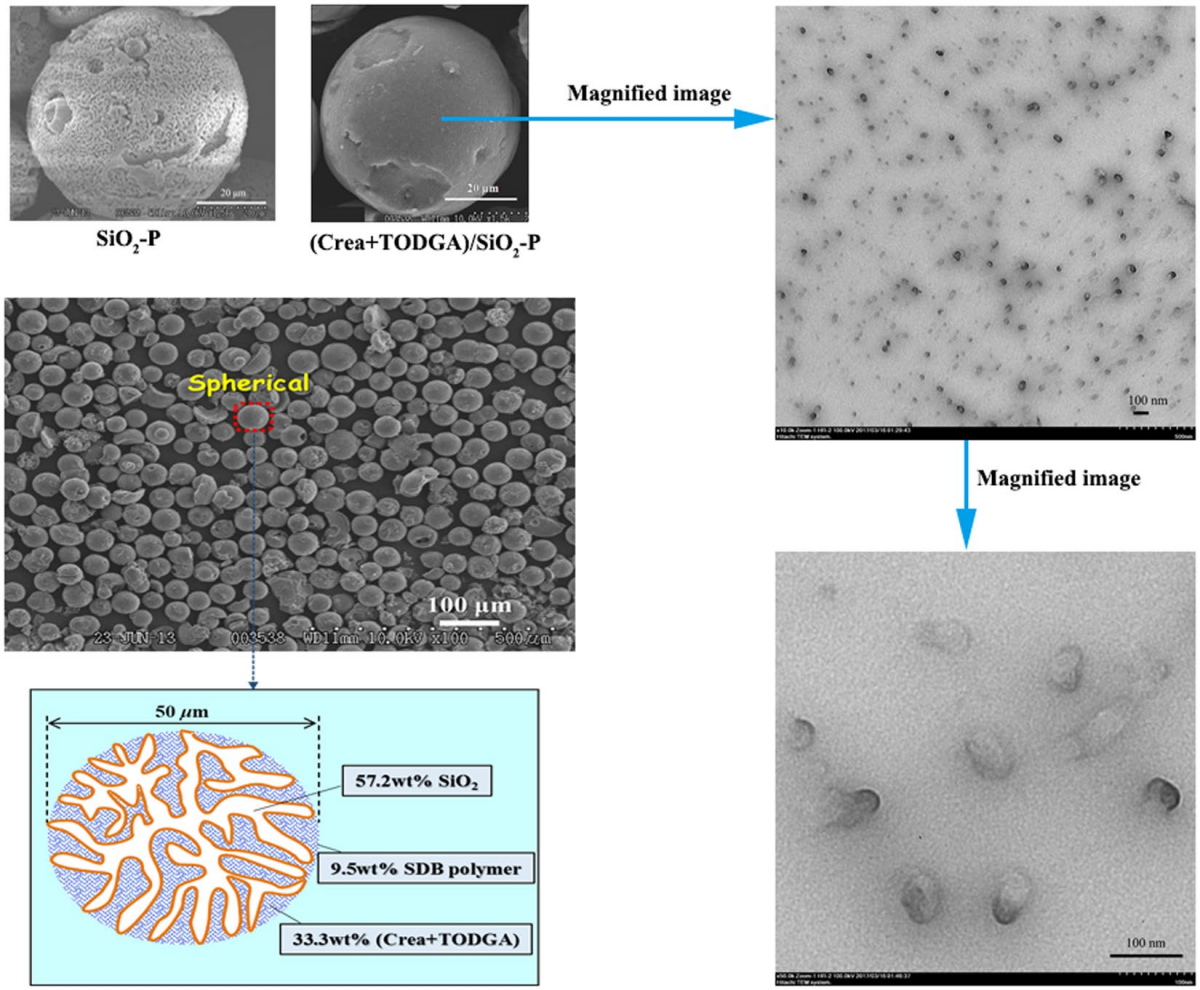
Magnification of TEM and SEM images of (Crea + TODGA)/SiO_2_-Padsorbent.

**Figure 3:**
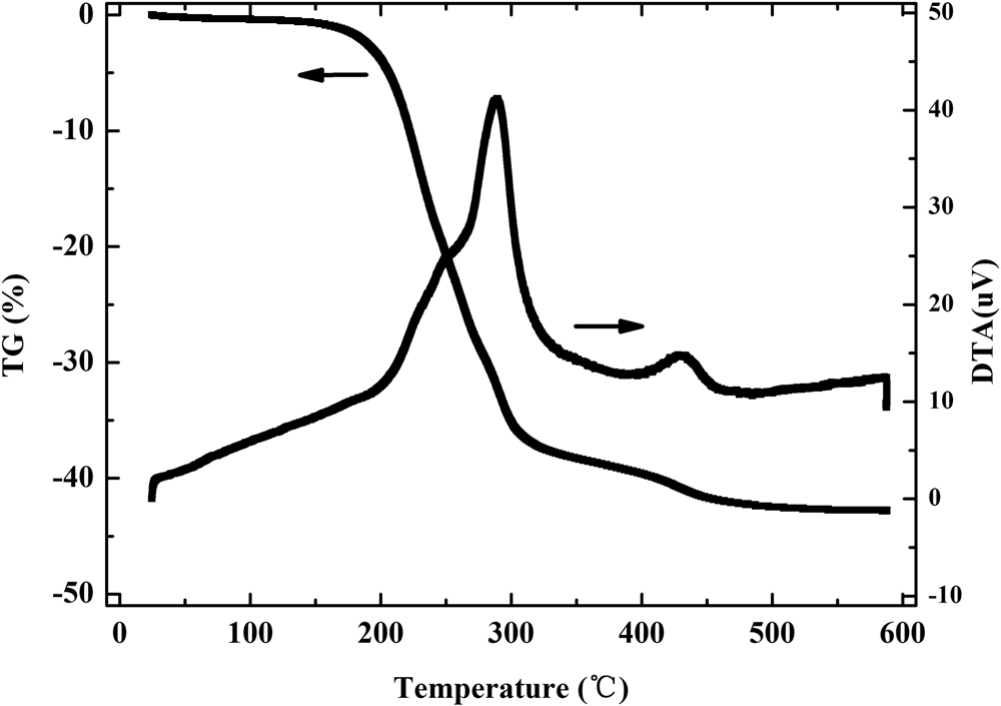
TG-DTA results of (Crea + TODGA)/SiO_2_-P adsorbent.

Figure 2 illustrates TEM and SEM images of synthesized (Crea + TODGA)/SiO_2_-P adsorbent. The spherical porous structure with a diameter of 50 µm which was close to the diameter of SiO_2_-P particles was confirmed. Surface with porous structures can be confirmed by the TEM and SEM images of adsorbent. And the smooth surface of (Crea + TODGA)/SiO_2_-P adsorbent without extractants accumulation indicated that Crea and TODGA were impregnating into the pores of porous SiO_2_-P support. The thermal stability of (Crea + TODGA)/SiO_2_-P adsorbent was evaluated by TG-DTA and results were showed in Fig. 3. Adsorbent composition (wt%) was calculated according to the TG-DTA results of SiO_2_-P particles and (Crea + TODGA)/SiO_2_-P adsorbent as follows: 33.3 wt% (Crea + TODGA), 9.5 wt% SDB copolymer and 57.2 wt% SiO_2_ substrate.

### Adsorption equilibrium experiment

**Figure 4:**
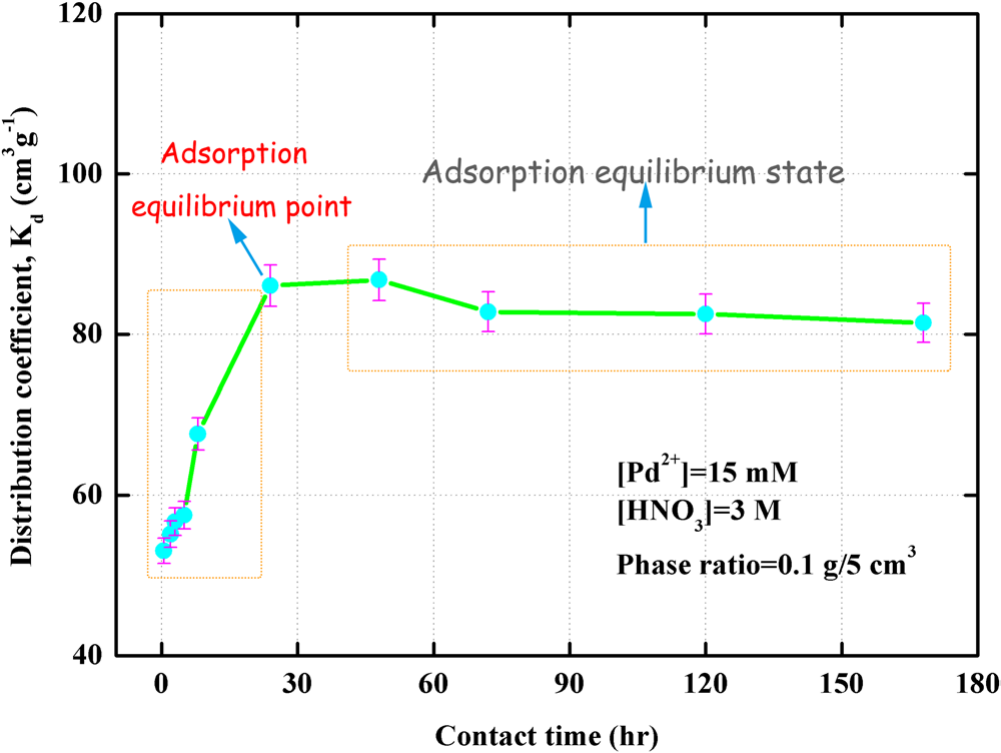
Effect of contact time on the adsorption of Pd(II) towards (Crea + TODGA)/SiO_2_-P adsorbent at 298 K.

To evaluate adsorption affinity of Pd(II) onto (Crea + TODGA)/SiO_2_-P adsorbent in nitric acid solution, effect of contact time on adsorption was studied by batch experiment in 3 M HNO_3_ solution containing 15 mM Pd(II) at 298 K. It was performed at phase ratio of 0.1 g/5 cm^3^ and shaking speed of 160 rpm. The results are shown in Fig. 4.

Figure 4 shows the effect of contact time on the adsorption of Pd(II) towards (Crea + TODGA)/SiO_2_-P adsorbent at 298 K. As can be seen, the adsorption of Pd(II) towards (Crea + TODGA)/SiO_2_-P adsorbent increased significantly with the increase of contact time from 0.5 to 24 hr and basically kept constant value from 24 to 48 hr. The distribution coefficient (*K*_d_) of Pd(II) was 53.09 cm^3^/g at 0.5 hr, 55.14 cm^3^/g at 2 hr, 56.70 cm^3^/g at 3 hr, 57.52 cm^3^/g at 5 hr, 67.64 cm^3^/g at 8 hr, 86.08 cm^3^/g at 24 hr, 86.80 cm^3^/g at 48 hr. The results indicated that the contact time to reach equilibrium state was around 24 hr under experiment condition. Pd(II), one of representative fission elements in HLLW solution, showed quick adsorption kinetics onto the novel (Crea + TODGA)/SiO_2_-P adsorbent. It resulted from the effective complexation of Pd(II) with extractants Crea and TODGA inside (Crea + TODGA)/SiO_2_-P adsorbent. And Crea, with N, O, S three atoms constituting space macrocyclic structure, may have accurately selective adsorption for Pd(II) (Eq. 2). In addition, the distribution coefficient (*K*_d_, cm^3^/g) values of Pd(II) decreased slightly with the increase of contact time from 48 to 72 hr at 298 K. The distribution coefficient (*K*_d_) of Pd(II) was 82.81 cm^3^/g at 72 hr, 82.54 cm^3^/g at 120 hr. Such a change in the trend of distribution coefficient might originate from the water-soluble degraded compounds of extractants Crea and TODGA contained in the adsorbent after contacting with HNO_3_ solution for a long contact time.

2 $$P{{\rm{d}}}^{2+}+2N{O}_{3}^{-}+(Crea+TODGA)/Si{O}_{2}-P\to P{\rm{d}}{(NO{}_{3})}_{2}\,\bullet \,(Crea+TODGA)/Si{O}_{2}-P$$

### Nitric acid effect on adsorption behavior of (Crea + TODGA)/SiO_2_-P

**Figure 5:**
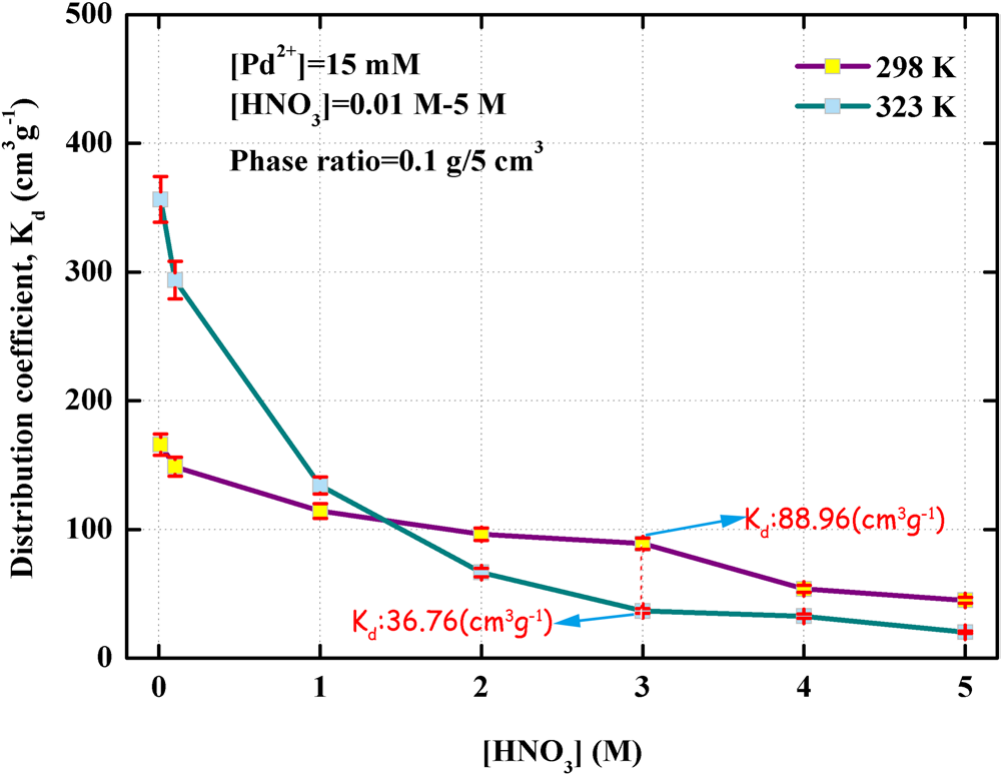
Effect of HNO_3_ concentration on the adsorption of Pd(II) towards (Crea + TODGA)/SiO_2_-P adsorbent at 298 K and 323 K.

During partitioning process of spent nuclear fuel, the various concentrations of HNO_3_ were usually used as the conventional solvent to dissolve multiple metals. Since (Crea + TODGA)/SiO_2_-P adsorbent exhibited strong selective adsorption to Pd(II), resistance properties of adsorbent against nitric acid was examined subsequently. To estimate the relationship between the adsorption behavior of (Crea + TODGA)/SiO_2_-P and HNO_3_ concentrations, a weighed amount of adsorbent and the different concentrations of HNO_3_ solution containing 15 mM Pd(II) were mixed in a glass vial, then shaken for 24 hr to reach adsorption equilibrium state at 298 K and 323 K. The adsorption behavior of Pd(II) towards (Crea + TODGA)/SiO_2_-P adsorbent in the range of 0.01–5 M HNO_3_ were illustrated in Fig. 5.

Figure 5 shows the effect of HNO_3_ concentration on the adsorption of Pd(II) towards (Crea + TODGA)/SiO_2_-P at 298 K and 323 K. As can been seen, the distribution coefficients *K*_d_ of Pd(II) decreased gradually as the HNO_3_ concentrations increased from 0.01–5 M at 298 K. The distribution coefficient (*K*_d_) of Pd(II) was 165.8 cm^3^/g in 0.01 M HNO_3_ solution, 148.7 cm^3^/g in 0.1 M HNO_3_ solution, 114.3 cm^3^/g in 1 M HNO_3_ solution, 96.28 cm^3^/g in 2 M HNO_3_ solution, 88.96 cm^3^/g in 3 M HNO_3_ solution, 53.81 cm^3^/g in 4 M HNO_3_ solution, 44.96 cm^3^/g in 5 M HNO_3_ solution. It is indicated that HNO_3_ concentrations had a notable influence on the stability and the adsorption ability of (Crea + TODGA)/SiO_2_-P adsorbent towards Pd(II). According previous report, the ability of Pd(II) to form nitrato-complexes is relatively low, increasing the concentration of HNO_3_ does not promote its adsorption onto resins with a weak-base group^21^. The other reason is perhaps that the competing extraction of Pd(II) nitrate complexes species with H^+^ in the adsorption leads to the decrease of *K*_d_ with increasing HNO_3_ concentration. Namely, the adsorption of (Crea + TODGA)/SiO_2_-P for Pd(II) decreased with the decreasing of the HNO_3_ concentration which was caused by the protonation of (Crea + TODGA) through association of oxygen atom with HNO_3_ via hydrogen bonding (Eq. 3). Compared with the adsorption of behavior of Pd(II) in different HNO_3_ concentration range at 298 K, the *K*_d_ values at 323 K larger than that at 298 K in low HNO_3_ concentration solution. The distribution coefficient (*K*_d_) of Pd(II) was 356.3 cm^3^/g, 293.6 cm^3^/g and 123.2 cm^3^/g in 0.01 M, 0.1 M and 1 M HNO_3_ solution, respectively. It can be concluded that higher temperature may activate the cations moving faster for enhancing adsorption. However, the adsorption ability of (Crea + TODGA)/SiO_2_-P adsorbent dramatically declined under the effect of in high acidity solution at 323 K. The distribution coefficient (*K*_d_) of Pd(II) was 66.58 cm^3^/g, 36.76 cm^3^/g, 32.60 cm^3^/g and 20.26 cm^3^/g in 2 M, 3 M, 4 M and 5 M HNO_3_ solution, respectively. Such a low distribution coefficient (*K*_d_) indicated that extracting agent structure was destroyed so that numbers of effective adsorption activity site reduced under the effect of high temperature and high acidity.

3 $$HN{O}_{3}\,+(Crea+TODGA)/Si{O}_{2}-P\to HNO{}_{3}\,_{\bullet }^{\,}(Crea+TODGA)/Si{O}_{2}-P$$

**Figure 6:**
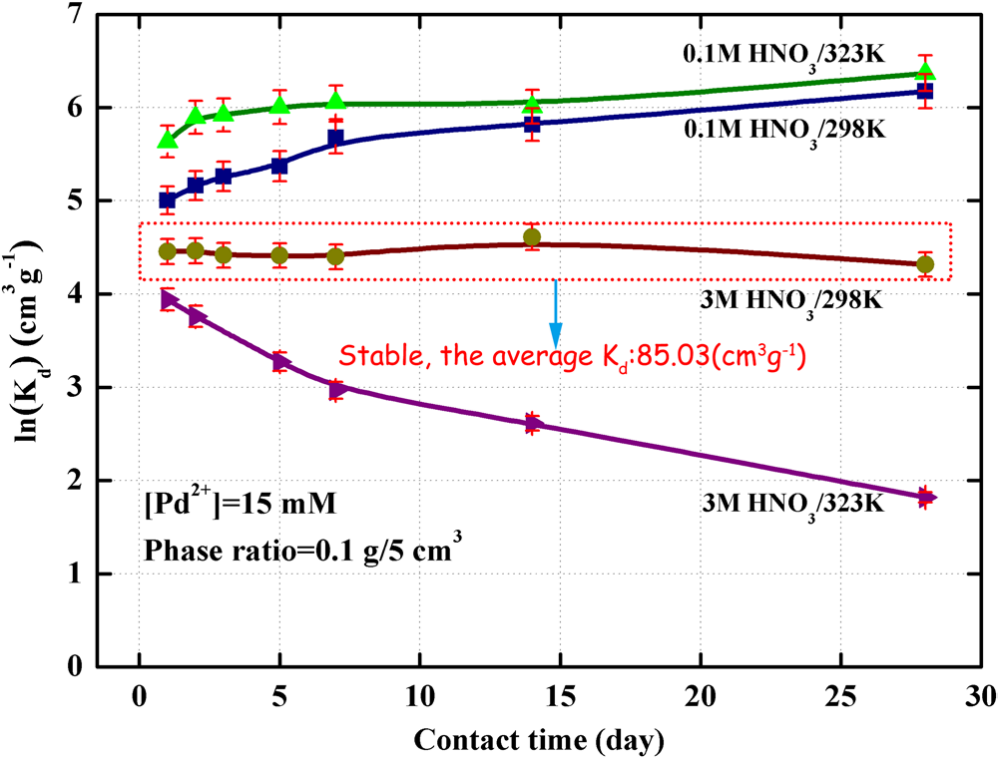
Effect of contact time on the adsorption of Pd(II) towards (Crea + TODGA)/SiO_2_-P adsorbent.

Considering of the service life of adsorbent, effect of contact time on the adsorption of Pd(II) towards (Crea + TODGA)/SiO_2_-P adsorbent in 0.1 M and 3 M HNO_3_ was observed at temperature 298 K and 323 K to further understand the resistant behavior of (Crea + TODGA)/SiO_2_-P adsorbent against nitric acid. The contact time was from 1 day to 28 days and the phase ratio was 0.1 g/5 cm^3^, respectively. The results were illustrated in Fig. 6.

Figure 6 shows the effect of contact time on the adsorption of Pd(II) towards (Crea + TODGA)/SiO_2_-P adsorbent. In 0.1 M HNO_3_ solution, adsorption of Pd(II) on (Crea + TODGA)/SiO_2_-P adsorbent increased with contact time increase from 1 day to 28 days which showed similar adsorption behavior at 298 K and 323 K. The *K*_d_ values of Pd(II) at 28 days were 480.2 cm^3^/g at 298 K and 582.7 cm^3^/g at 323 K, respectively. Such a high *K*_d_ value at 28 days indicated that the structure of (Crea + TODGA)/SiO_2_-P adsorbent was stable in 0.1 M HNO_3_ solution. In addition, higher temperature was beneficial to the adsorption of (Crea + TODGA)/SiO_2_-P for Pd(II) through promoting the movement of ions. In contrast to the adsorption for Pd(II) in 0.1 M HNO_3_ solution, the *K*_d_ values in 3 M HNO_3_ were lower because of the competition of Pd(II) and H^+^ for adsorbing. In 3 M HNO_3_ solution, the adsorption of Pd(II) at 298 K basically kept a constant state with an increase in contact time. And the average distribution coefficient (*K*_d_) of Pd(II) was 85.03 cm^3^/g which indicated (Crea + TODGA)/SiO_2_-P can be proposed as an acceptable absorbent for separation of Pd(II) in 3 M HNO_3_ at 298 K. However, the effect of contact time on adsorption of Pd(II) onto the adsorbent at 323 K was obvious. When the contact time lasted from 1 day to 28 days, the *K*_d_ value of Pd(II) decreased from 51.52 to 6.170 cm^3^/g. It indicated that the extractants Crea and TODGA were out of activity by contacting with nitric acid for a long time under high temperature.

### Decomposition of adsorbent

In previous study, the adsorption ability of (Crea + TODGA)/SiO_2_-P adsorbent for Pd(II) was obviously reduced under the effect of the high temperature and high HNO_3_ concentration. And the extractants Crea and TODGA which were organic material usually suffer damages from strong acid and high temperature. To further confirm the acid resistance of the (Crea + TODGA)/SiO_2_-P adsorbent, degradation of the adsorbent was investigated by analysis of dissolved organic carbon in liquid phase made by decomposition of the impregnated Crea and TODGA.

**Figure 7:**
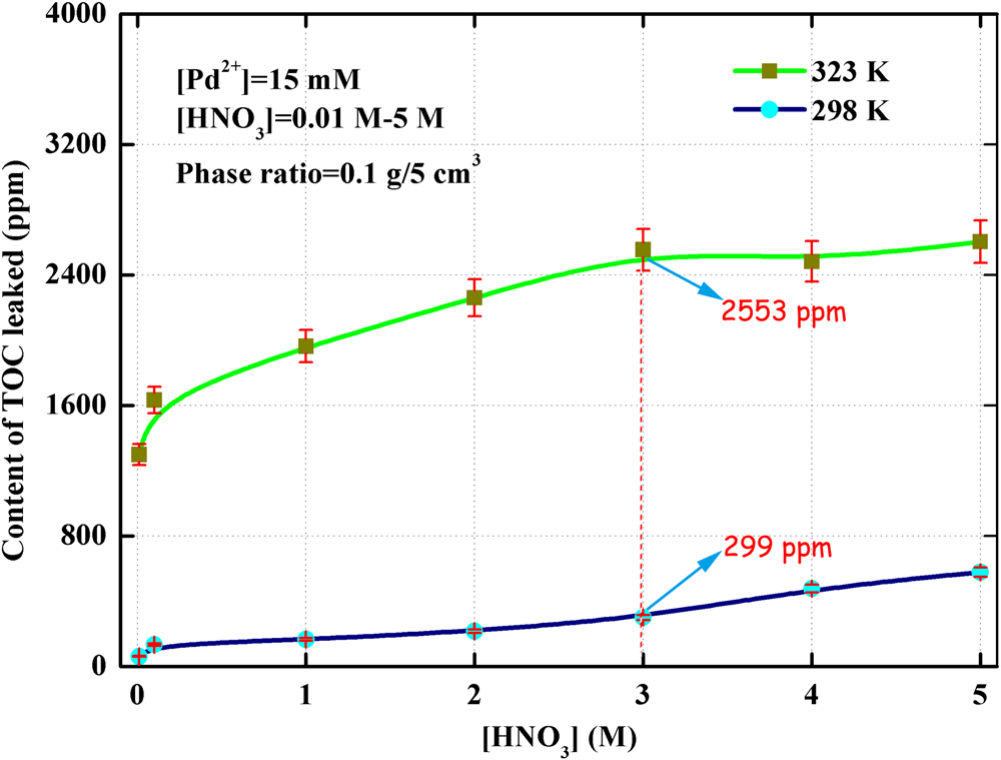
Effect of HNO_3_ concentration on the content of TOC leaked from (Crea + TODGA)/SiO_2_-P adsorbent.

Figure 7 shows the leakage of TOC from (Crea + TODGA)/SiO_2_-P adsorbent in the range of 0.01 M-5 M HNO_3_ at 298 K and 323 K, respectively. It can be seen that the content of TOC leaked from (Crea + TODGA)/SiO_2_-P adsorbent increased with the increasing of HNO_3_ concentration. At 298 K, the bleeding of TOC was from 61.17 ppm to 577.3 ppm with the HNO_3_ concentration from 0.01 M to 5 M. Meanwhile, the leakage values at 323 K were obviously higher than those at 298 K under experiment condition. At 323 K, the bleeding of TOC was from 1298 ppm to 2602 ppm with the HNO_3_ concentration from 0.01 M to 5 M. The maximum leakage percent was 57.86% in 5 M HNO_3_ at 323 K. This indicated that (Crea + TODGA)/SiO_2_-P seriously deteriorated upon contacting with high concentration of HNO_3_ solution at high temperature. The results are consistent with the adsorption ability of (Crea + TODGA)/SiO_2_-P at 298 K and 323 K. Consideration of the limited quantity of extractant leaked, the (Crea + TODGA)/SiO_2_-P adsorbent which showed good stability against nitric acid at 298 K was thought as an acceptable adsorbent.

Considering of the economic costs and the lifespan of adsorbent, the content of TOC leaked from (Crea + TODGA)/SiO_2_-P adsorbent after shaking in 0.1 M and 3 M HNO_3_ solution with the change of contact time under the liquid-solid ratio of 50 cm^3^/g at 298 K and 323 K were measured by total organic carbon TOC analyzer.

**Figure 8:**
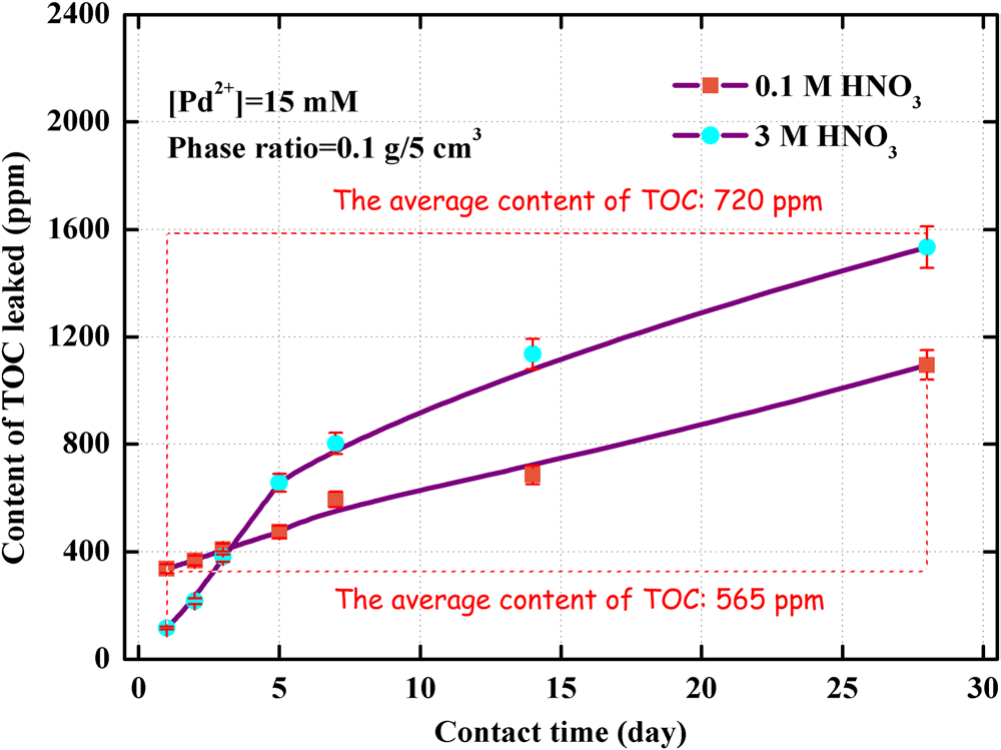
Effect of contact time on the content of TOC leaked from (Crea + TODGA)/SiO_2_-P adsorbent in 0.1 M and 3 M HNO_3_ solution at 298 K.

**Figure 9:**
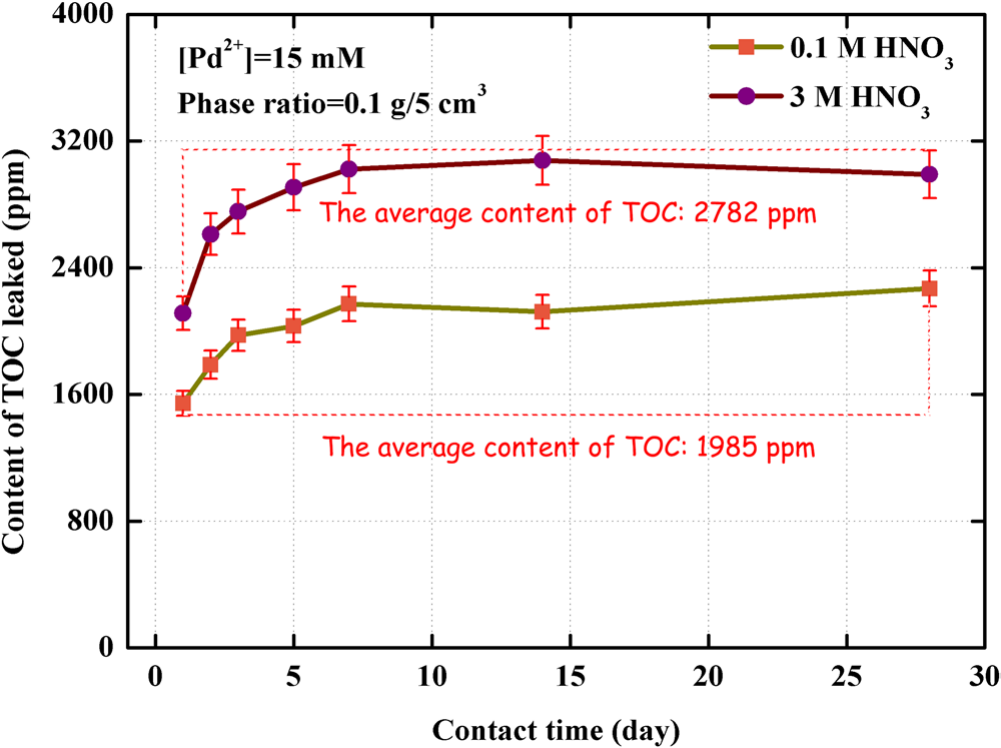
Effect of contact time on the content of TOC leaked from (Crea + TODGA)/SiO_2_-P adsorbent in 0.1 M and 3 M HNO_3_ solution at 323 K.

Figure 8 showed the effect of contact time on the content of TOC leaked from (Crea + TODGA)/SiO_2_-P adsorbent in 0.1 M and 3 M HNO_3_ solution at 298 K. The concentration of extractant leaching to the solution increased with an increase of the contact time within 28 days at 298 K. The average amount of TOC in contact time range of 1–28 days in 0.1 M HNO_3_ solution was 565.9 ppm which was lower that a range of 115.7–1533 ppm with an average of 720.4 ppm leaked in 3 M HNO_3_ solution. The acidity in HLLW is usually around 3.0 M HNO_3_ in reprocessing of nuclear spent fuel. Consideration of the limited quantity of extractant leaked, the (Crea + TODGA)/SiO_2_-P adsorbent was thought as an adsorbent possessing good acid-resistant ability at 298 K in 3 M HNO_3_ solution. Figure 9 showed the leakage behavior of carbon of the absorbent in 0.1 M and 3 M HNO_3_ with contact time from 1 day to 28 days at 323 K. To compare the results of Figs 8 and 9, the TOC values at 323 K were obviously higher than those at 298 K at all contact time no matter in 0.1 M or 3 M HNO_3_. Namely, the leakage of TOC from (Crea + TODGA)/SiO_2_-P absorbent increased noticeably with an increase in temperature, especially at high temperature and high HNO_3_ concentration. The influence of low temperature on the stability of (Crea + TODGA)/SiO_2_-P in low HNO_3_ concentration was basically permissible under experimental conditions.

## Conclusion

For separation Pd(II) from high level liquid waste (HLLW), a new kind of silica-based (Crea + TODGA)/SiO_2_-P absorbent was prepared by impregnation of Crea and TODGA two extractants into SiO_2_-P support with a mean diameter of 50 µm. To examine the applicability of (Crea + TODGA)/SiO_2_-P adsorbent, the adsorption properties of Pd(II) towards (Crea + TODGA)/SiO_2_-P adsorbent was evaluated in 3 M HNO_3_ at 298 K. Adsorbent showed good adsorption affinity for Pd(II) and reached equilibrium state within 24 hr. Then, the nitric acid resistances of (Crea + TODGA)/SiO_2_-Pabsorbent and the degradation of the absorbent were investigated by batch methods. The adsorption ability of (Crea + TODGA)/SiO_2_-P adsorbent for Pd(II) and the content of TOC leaked decreased with increasing of HNO_3_ concentration. The HNO_3_ concentrations had a certain influence on the adsorption ability of (Crea + TODGA)/SiO_2_-P adsorbent due to the protonation of (Crea + TODGA)/SiO_2_-P which was a competitive adsorption reaction to Pd(II). In 3 M HNO_3_, the average of *K*_d_ value in contact time range of 1–28 days was 85.03 cm^3^/g and 26.10 cm^3^/g at 298 K and 323 K, respectively. And the content of TOC leaked from the adsorbent at 28 days were 1095 ppm and 2989 ppm at 298 K and 323 K, respectively. Means, structure of (Crea + TODGA)/SiO_2_-P seriously decomposed upon contacting with high concentration of HNO_3_ solution at high temperature. But the acid resistant behavior of (Crea + TODGA)/SiO_2_-P adsorbent at 298 K was acceptable. From the results in this study, (Crea + TODGA)/SiO_2_-P can be proposed as an applicable and efficient absorbent for separation of Pd(II) in 3 M HNO_3_ at 298 K.

## Electronic supplementary material

Supplementary Information
