# Supplementary material for: Effect of HNO3 concentration on a novel silica-based adsorbent for separating Pd(II) from simulated high level liquid waste
Source: Sci Rep. 2017 Sep 12;7:11290. doi: 10.1038/s41598-017-11879-6 (PMC5595992; doi:10.1038/s41598-017-11879-6)
Supplement: Supplementary file 1 — Supplementary Information [file 41598_2017_11879_MOESM1_ESM.pdf]

Title:

Effect of HNO_3 concentration on a novel macroporous silica-based adsorbent for separating Pd(II) from simulated high level liquid waste

Running title:

A novel macroporous silica-based adsorbent for Pd(II)

Authors:

Guo Ge, XuYuanlai*, Yang Xinxin, Wang Fen, Zhou Fang, Yu Junxia, Chi Ruan

Authors affiliations:

Key Laboratory for Green Chemical Process of Ministry of Education, Hubei Key Laboratory of Novel Reactor and Green Chemical Technology, Wuhan Institute of Technology, Wuhan, 430073, China

*Correspondence author

Dr. Xu Yuanlai

Key Laboratory for Green Chemical Process of Ministry of Education

Wuhan Institute of Technology

693 Xiongchu Street, Wuhan, 430073, Hubei, PR China

E-mail: xuyuanlai@163.com

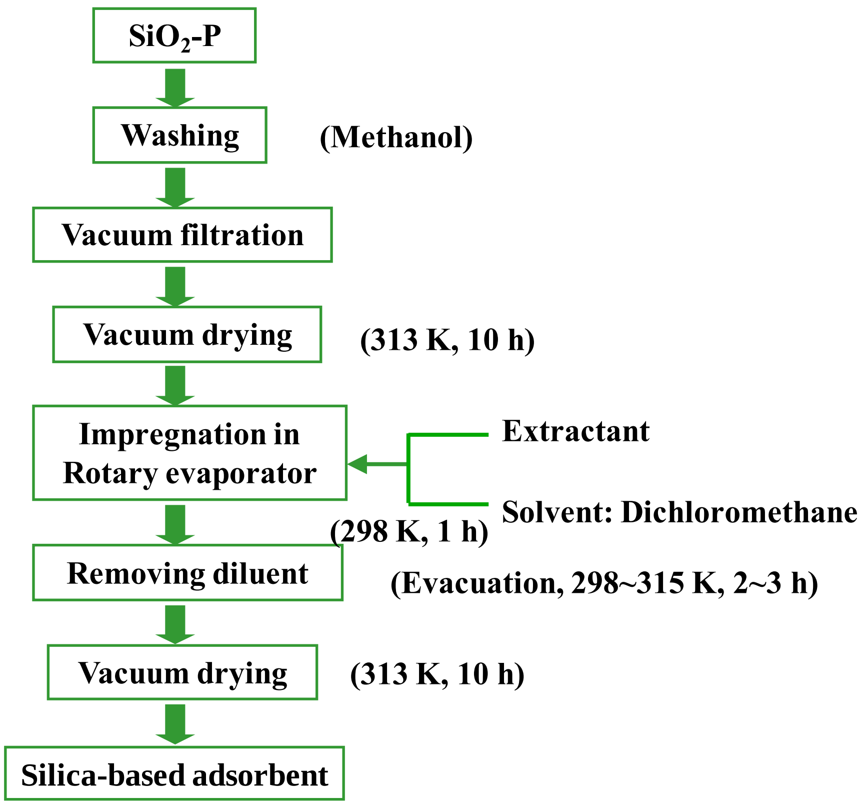

Supplementary Figure 1. Synthesized method of macroporous silica-based adsorbent (Crea+TODGA)/SiO_2-P by impregnating two of the extractants Crea (N'-N'-di-n-hexyl-thiodiglycolamide) and TODGA (N, N, N', N'-tetraoctyl-3-oxapentane1, 5-diamide) into SiO_2-P support.
